# NUFFT-Based Iterative Image Reconstruction via Alternating Direction Total Variation Minimization for Sparse-View CT

**DOI:** 10.1155/2015/691021

**Published:** 2015-05-18

**Authors:** Bin Yan, Zhao Jin, Hanming Zhang, Lei Li, Ailong Cai

**Affiliations:** China National Digital Switching System Engineering and Technological Research Center, Zhengzhou 450002, China

## Abstract

Sparse-view imaging is a promising scanning method which can reduce the radiation dose in X-ray computed tomography (CT). Reconstruction algorithm for sparse-view imaging system is of significant importance. The adoption of the spatial iterative algorithm for CT image reconstruction has a low operation efficiency and high computation requirement. A novel Fourier-based iterative reconstruction technique that utilizes nonuniform fast Fourier transform is presented in this study along with the advanced total variation (TV) regularization for sparse-view CT. Combined with the alternating direction method, the proposed approach shows excellent efficiency and rapid convergence property. Numerical simulations and real data experiments are performed on a parallel beam CT. Experimental results validate that the proposed method has higher computational efficiency and better reconstruction quality than the conventional algorithms, such as simultaneous algebraic reconstruction technique using TV method and the alternating direction total variation minimization approach, with the same time duration. The proposed method appears to have extensive applications in X-ray CT imaging.

## 1. Introduction

X-ray computed tomography (CT) has been widely used for imaging applications in various fields, such as industrial nondestructive testing [1] and medical diagnosis [2], for its advantages of noninvasive and high spatial resolution. However, in many practical applications of X-ray computed tomography, complete projection data set cannot be obtained because of the limitation of scanning time, space, dose, and so on. Therefore, sparse angle scanning scheme is adopted to tackle these problems. On one hand, this scheme can speed up the scanning rate and decrease the X-ray radiation dose, such as breast and vascular imaging [3–6]. On the other hand, sparse data sampling can save much scanning time and it is of practice value when high reconstruction precision is not that urgent. To solve the sparse-view reconstruction problem, the classical methods should be upgraded, and a new algorithm needs to be developed.

Given the unsatisfactory Tuy-Smith condition [7, 8] in sparse-view, a CT image cannot be accurately reconstructed via analytic method. To solve the ill-posed problem [9, 10], numerous iterative algorithms [11–13] have been proposed based on spatial domain. However, these iterative algorithms are time-consuming and have a great demand for hardware resources. Despite applying hardware speedup technology, such as an ordinary graphics processing unit [14], these algorithms still consume a considerable amount of time.

Compressive sensing theory by Candés et al. [15–17] provided a new idea for the exact recovery of an image from the sparse samples of its discrete Fourier transform. The exact reconstruction relies on the assumption that there exists sparse representation for an image. A number of cases are known to have sparse gradient-magnitude images. In some cases, minimizing the total variation (TV) can generate accurate images from sparse samples [18–20]. Therefore, combining TV regularization with the iterations of the simultaneous algebraic reconstruction technique (SART), hereinafter called SART–TV [21], can improve reconstruction image quality while decreasing mean-squared error. Based on the projection onto the convex sets (POCS) algorithm, the adaptive steepest descent-POCS (ASD-POCS) algorithm [20] can effectively handle incomplete datasets and demonstrates excellent performance in sparse-view CT applications.

The rapid increase in the size of scanning data has highlighted the importance of reducing reconstruction time and improving reconstruction quality. It is known that processing the same signal in the frequency space is faster than that in the spatial domain by fast Fourier transform (FFT). Several algorithms for image reconstruction in the frequency space can also be developed on the basis of fast Fourier transform. Several studies have been conducted to achieve this goal. In 1981, Stark et al. [22] developed direct Fourier methods (DFM) using central slice theorem and obtained favorable results. In 2003, Seger and Danielsson [23] analyzed the missing projection data in the frequency domain and proposed a reconstruction method for the scanned timber data according to Fourier transform. In 2013, Fahimian et al. [24] presented a Fourier-based iterative reconstruction in medical X-ray CT, and numerical experiment results showed that this method required less computation time than other iterative algorithms. These achievements facilitated the development of an improved algorithm for solving the sparse-view reconstruction problem in the frequency domain.

Because of the limitation of FFT, that is, its unsuitability for application to nonuniform samples, this technique requires further enhancement to improve its universality. To this end, nonuniform FFT (NUFFT) [25] has been recently developed to overcome this limitation without increasing the computation complexity of FFT. NUFFT is also basis of the proposed reconstruction algorithm in this study. Motivated by the feature of NUFFT for data distribution, some approaches have been proposed to reconstruct a CT image to deal with frequency data. In 2004, Matej et al. [26] proposed an iterative tomographic image reconstruction method using NUFFT and obtained better results with this technique than with the filtered back-projection (FBP) algorithm. In 2006, Zhang-O'Connor and Fessler [27] proposed Fourier-based forward- and back-projectors for fan-beam tomographic image reconstruction. However, these proposed NUFFT cannot effectively solve the problems in sparse-view image reconstruction. The NUFFT just was especially applied as a transition during iteration in spatial domain, which in turn burdened computation consumption.

In this paper, our study aims to present a promising contribution to the task of image reconstruction from sparse-view by combining the alternating direction total variation minimization (ADTVM) technique with NUFFT to establish a new method which is suitable for large-scale reconstruction because of its low computational requirement. The algorithm is developed under the framework of alternating direction method (ADM) which shows high efficiency and stability. The advantages of the proposed algorithm are verified by the results of several groups of experiments.

The organization of this paper is organized as follows.  Section 1 concisely reviews the basic CT reconstruction and the state of the art of sparse-view image reconstruction.  Section 2 describes the basic principles of the proposed method, including the reconstruction model and the corresponding algorithm based on NUFFT and ADTVM.  Section 3 demonstrates groups of typical experiments results, including numerical simulation and real data ones.  Section 4 discusses the findings of the experiments and concludes the paper.

## 2. Method and Material

### 2.1. Image Reconstruction Model

In this work, we consider temporarily parallel geometry. In parallel geometry, 2D function *f* (objection function) is defined in a compact support of spatial domain, which means that it vanishes outside a finite region of the plane. In the (*x*, *y*) plane, the general formation for the line integral, known as the Radon transform of *f*(*x*, *y*), is

(1) $$\begin{array}{c} p_{\theta }\left(s\right)=\overset{\infty }{\underset{-\infty }{\iint }}\;f\left(x,y\right)\delta \left(x\cos\theta +y\sin\theta -s\right)dx\;dy. \end{array}$$

**Figure 1:**
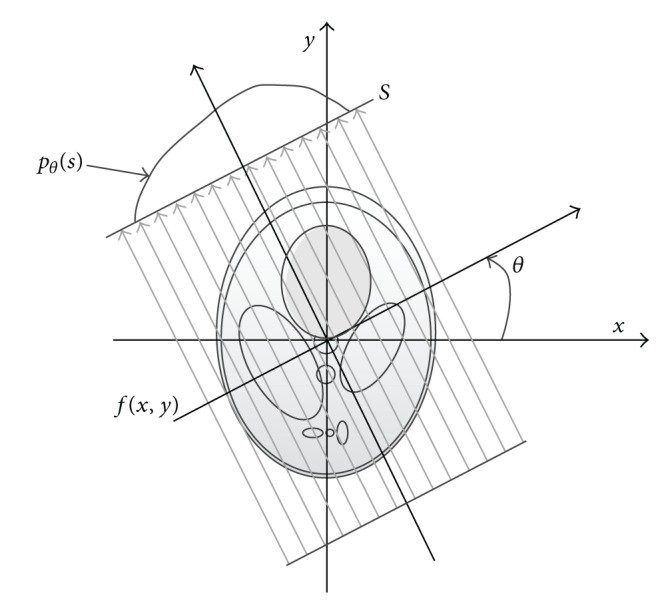
An object *f*(*x*, *y*) and its parallel projection *p*_*θ*_(*s*).

In Figure 1, projection *p*_*θ*_(*s*) consists of a collection of line integrals (1) taken along straight parallel lines in the plane that means a collection of *p*_*θ*_(*s*) with constant *θ* ∈ [0, *π*/2] and *s* ∈ [−*S*/2, *S*/2].

CT image reconstruction is an inverse problem, and the observed projections should be converted into images which reflect the distribution of the attenuation coefficient of the interested physical object. A conventional reconstruction method, that is, the direct Fourier methods (DFM), is established based on the Fourier slice theorem. Basically, the steps of DFM can be summarized as follows:

- 1D discrete Fourier transform of the parallel projections taken at different angles;
- polar to Cartesian grid interpolations;
- 2D inverse Fourier transform.

From the perspective of DFM, the observation equation in the Fourier domain can be expressed as follows:

(2) $$\begin{array}{c} P_{\theta }\left(\rho \right)=\overset{\infty }{\underset{-\infty }{\int }}p_{\theta }\left(s\right)e^{-j2\pi \rho s}ds, \end{array}$$

where *P*_*θ*_(*ρ*) is observed by the Fourier transform of the measured projection data *p*_*θ*_(*s*) and *ρ* is the frequency variable of Fourier transform. The process proceeds with the use of FFT and is characterized by high accuracy. According to Fourier central slice theory, the 1D FFT of the projection *P*_*θ*_(*ρ*) is equal to $\hat{f}(u,v)$, which is derived from the 2D FFT of the reconstructed image in a certain angle. Therefore, the relationship is described as follows:

(3) Pθρ=∫−∞+∞pθse−j2πρsds=f^u,vu=ρcos⁡⁡θv=ρsin⁡θ,

where $\hat{f}\left(u,v\right)=\iint_{-\infty }^{+\infty }f(x,y){\text{e}}^{-j2\pi (xu+yv)}dx\;dy$. The equation shows an obvious equivalence corresponding to frequency projection *P*_*θ*_(*ρ*) with $\hat{f}(u,v)$ in polar coordinates.

To avoid interpolation errors, such as DFM in the image and frequency domains, this study introduces NUFFT, which can translate polar coordinates into the image space without interpolation. This technique can significantly improve the accuracy of reconstruction. Let *F*_*N*_ represent the NUFFT operator, such that the following equation can be derived:

(4) FNf=∬−∞+∞fx,ye−j2πxu+yvdx dyu=ρcos⁡⁡θv=ρsin⁡θ=∫−∞∞pθse−j2πρsds=Pθρ.

The reconstruction module can be discretely shown as follows:

(5) $$\begin{array}{c} P=F_{N}f, \end{array}$$

where the (observed) constant *F*_*N*_ and the variant **f** are the vector forms of Fourier sampling and objection function, respectively. Matrix *F*_*N*_ stands for the NUFFT of **f**. The Fourier transform *F*_*N*_ **f** in (5) and its adjoint *F*_*N*_^*H*^ **f** can be implemented by using FFT to generate a fast and accurate evaluation.

In sparse-view reconstruction, (5) is ill-posed, and the projection data are insufficient for exact reconstruction. Mathematically, the problem that we consider here involves insufficient data, such that (5) is underdetermined. To solve this linear and underdetermined equation, we specify a TV minimization algorithm that considers the reconstruction to be a task of finding the best solution to the following optimization problem:

(6)  f∗=arg min⁡⁡fTV, subject  to  s.t. P=FNf, f≥0,

where ‖**f**‖_TV_ denotes the discretization of the TV term and ‖**f**‖_TV_ = ∑_*i*_‖*D*_*i*_ **f**‖_1_. By applying the directional gradients operators *D*_*i*_ [20, 28], model (6) can also be written as follows:

(7) min⁡ ∑iwi1+λ2FNf−P22,s.t. 00Dif=wi, f≥0,

where *λ* is the fidelity parameter to control the data consistency in the object function.

**Figure 2:**
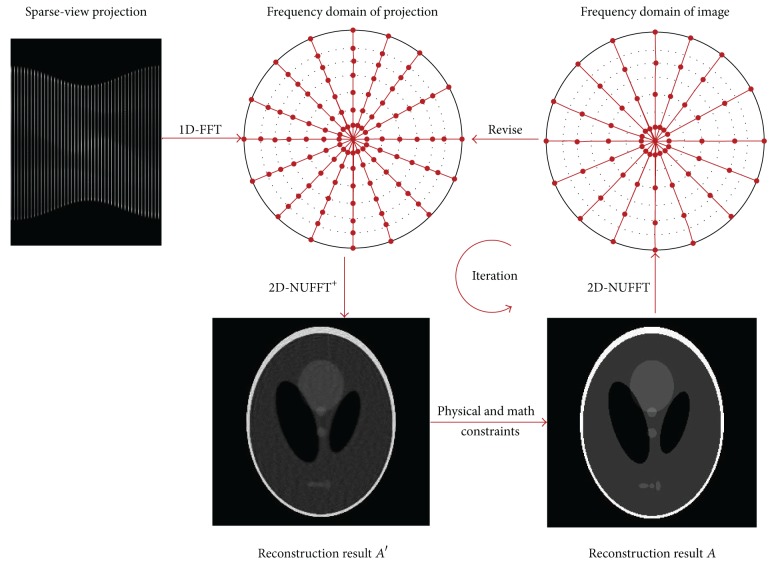
The flowchart of sparse-view image reconstruction for the model.

Therefore, the overall reconstruction flowchart can be summarized as Figure 2.

### 2.2. NUFFT with ADM for the Model

The above constrained optimization is addressed by converting the equation into its unconstrained form by applying the augmented Lagrange function:

(8) min⁡⁡ Lf,wi=∑iρi2wi1+uiTDif−wi000000+ρi2Dif−wi22 +λ2FNf−P22,

where *ρ*_*i*_ is a scalar that denotes the penalty coefficient and *u*_*i*_ denotes the multipliers. The minimization processes with respect to variables **f** and **w**_*i*_ cannot be easily realized simultaneously by directly performing the optimization. Moreover, decomposing the variables by using ADM has a low computation cost. The ADM approach decouples the augmented Lagrange function into two subproblems, namely, the **w**-subproblem and the **f**-subproblem [29].

The **w**-subproblem can be written as follows:

(9) $$\begin{array}{c} \underset{}{\min}\;L_{f}\left(w_{i}\right)=u_{i}^{T}\left(D_{i}f-w_{i}\right)+\frac{\rho_{i}}{2}{\left\|D_{i}f-w_{i}\right\|}_{2}^{2}+{\left\|w_{i}\right\|}_{1}. \end{array}$$

The **w**-subproblem is separable with respect to **w**, and problem (9) can be efficiently solved by using the shrinkage operator [30], which is expressed as follows:

(10) $$\begin{array}{c} w_{i}^{*}=\max\left\{\left|D_{i}f+\frac{u_{i}}{\rho_{i}}\right|-\frac{1}{\rho_{i}},0\right\}\cdot \mathrm{sgn}\left(D_{i}f+\frac{u_{i}}{\rho_{i}}\right). \end{array}$$

In addition, with the aid of **w**_*i*_, the optimization of **f**-subproblem can be achieved by solving the following:

(11) min⁡⁡ Lwif=λ2FNf−P22 +∑iuiTDif−wi+ρi2Dif−wi22.

*L*_**w**_*i*__(**f**) is clearly a quadratic function, the gradient of which is expressed as follows:

(12) $$\begin{array}{c} l\left(f\right)=\lambda F_{N}^{H}\left(F_{N}f-P\right)+\underset{i}{\sum }\left(D_{i}^{T}u_{i}+\rho_{i}D_{i}^{T}\left(D_{i}f-w_{i}\right)\right). \end{array}$$

Force *l*(**f**) = 0 and the exact solution for *L*(**f**) is presented as follows:

(13) f∗=λFNTFN+∑iρiDiTDi+ ·λFNTP−∑iDiTuiT−ρiwi,

where *M* ^+^ denotes the Moore-Penrose inverse of matrix *M*. Theoretically, the exact minimizer can be used to solve the **f**-subproblem. However, the inverse or pseudo-inverse is too costly to compute numerically at each iteration. The augmented Lagrangian function (8) is expected to be minimized by solving the **w**-subproblem and the **f**-subproblem alternately. Therefore, solving the **f**-subproblem accurately at each sweep may be unnecessary. A robust and efficient nonmonotone alternating direction algorithm [31] is used to solve problem (13).

By using the solutions of **w**_*i*_^∗^ and **f** ^∗^, the multipliers are updated as follows:

(14) $$\begin{array}{c} u_{i}=u_{i}+\rho_{i}\left(D_{i}f^{*}-w_{i}^{*}\right). \end{array}$$

The optimized solution for (8) is attained by circularly applying (10) and (13) until *L*(**f**, **w**_*i*_) is minimized jointly with respect to (**f**, **w**_*i*_).

### 2.3. Algorithm of the Overall Framework

All issues in handling the subproblems have been addressed in Section 2.2. In light of all derivations presented above, the new algorithm for solving the reconstruction problem can be stated as follows.

**Algorithm 1 .**

Input projection data *p*, *λ*, *ρ*_*i*_ > 0. Initialize *u*_*i*_ = *u*_*i*_^(0)^ and starting points *w*_*i*_^0^, *u*_*i*_^0^ for all *i*. Set *k* = 0.

(1) make 1D FFT of *p*_*θ*_(*s*) with respect to *s*
  (15) $$\begin{array}{c} P_{\theta }\left(\rho \right)⟵\overset{+\infty }{\underset{-\infty }{\int }}p_{\theta }\left(s\right)e^{-j2\pi \rho s}ds; \end{array}$$
 
**while** “not achieved maximum iteration loops,”** Do**
(2) compute frequency domain *P*_*θ*_(*ρ*) via NUFFT;
(3) compute **f** by
  (16) fik+1⟵λFNTFN+∑iρiDiTDi+·λFNTP−∑iDiTuiT−ρiwi;
(4) compute **w** by
  (17) $$\begin{array}{c} w_{i}^{k+1}⟵\max\left\{\left|D_{i}f+\frac{u_{i}}{\rho_{i}}\right|-\frac{1}{\rho_{i}},0\right\}\cdot \mathrm{sgn}\left(D_{i}f+\frac{u_{i}}{\rho_{i}}\right); \end{array}$$
(5) update *u*_*i*_ by
  (18) $$\begin{array}{c} u_{i}^{k+1}⟵u_{i}+\rho_{i}\left(D_{i}f^{*}-w_{i}^{*}\right); \end{array}$$
(6) *k* ← *k* + 1
 
**End Do**

In this study, the proposed NUFFT reconstruction technique is developed on the basis of ADTVM. This technique is called NUFFT-ADM. According to the above algorithm, the proposed method demonstrates fast convergence and effective iteration through ADM. This method can be effectively implemented in large-scale reconstruction in sparse-view because of its low computational cost, thus making this technique promising in practical applications.

## 3. Experiments

To verify the performance of the proposed algorithm, both numerical simulations and real CT scan data experiments are conducted. All experiments are performed on an AMAX Tesla workstation with Intel Xeon E5520 dual-core CPU 2.27 GHz and 24 GB memory. All programs are performed using MATLAB 2011a. In all experiments, the parameter of TV is that primary penalty parameter *μ* and secondary penalty parameter *β* are 1024 and 32, respectively.

### 3.1. Numerical Phantom Simulation

The above algorithm is applied to validate its high efficiency. A group of 2D image reconstruction experiments are performed using a 2D Shepp-Logan phantom with a size of 256 × 256. This phantom is generated according to the definition of the ellipse phantom image. The scanning and reconstruction parameters in the experiment are listed in Table 1. The detector elements are equidistantly spaced 0.127 mm from one another.

**Figure 3:**
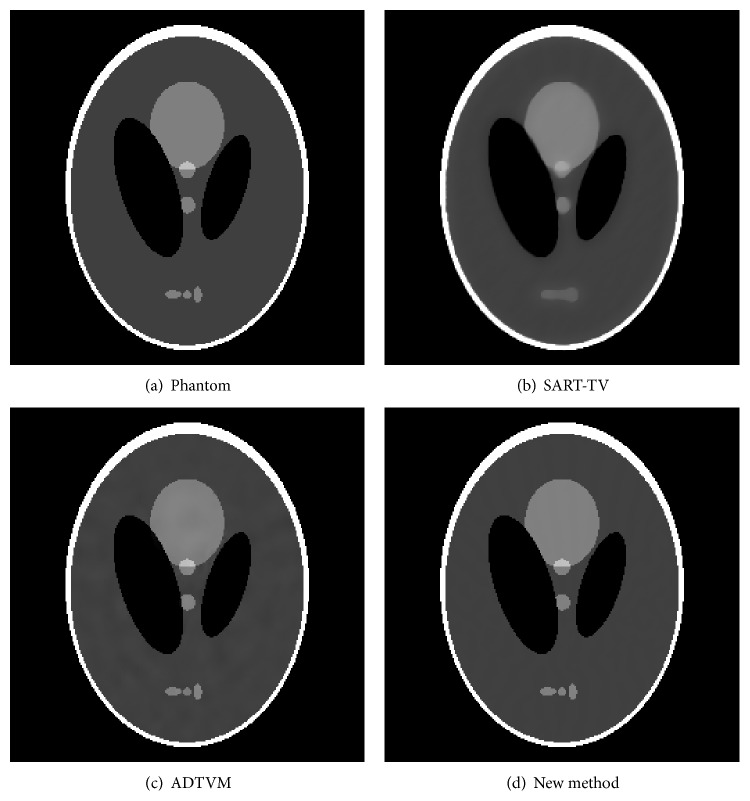
Image reconstructions of the Shepp-Logan phantom in 60-view scan. Display Window (0.1 0.5). (a) shows the original image. (b) shows the image after applying the SART-TV algorithm at 200 iterations. (c) gives the image after vua the ADTVM algorithm at 200 iterations. (d) presents the reconstruction image after using the NUFFT-ADM algorithm at 200 iterations.

**Figure 4:**
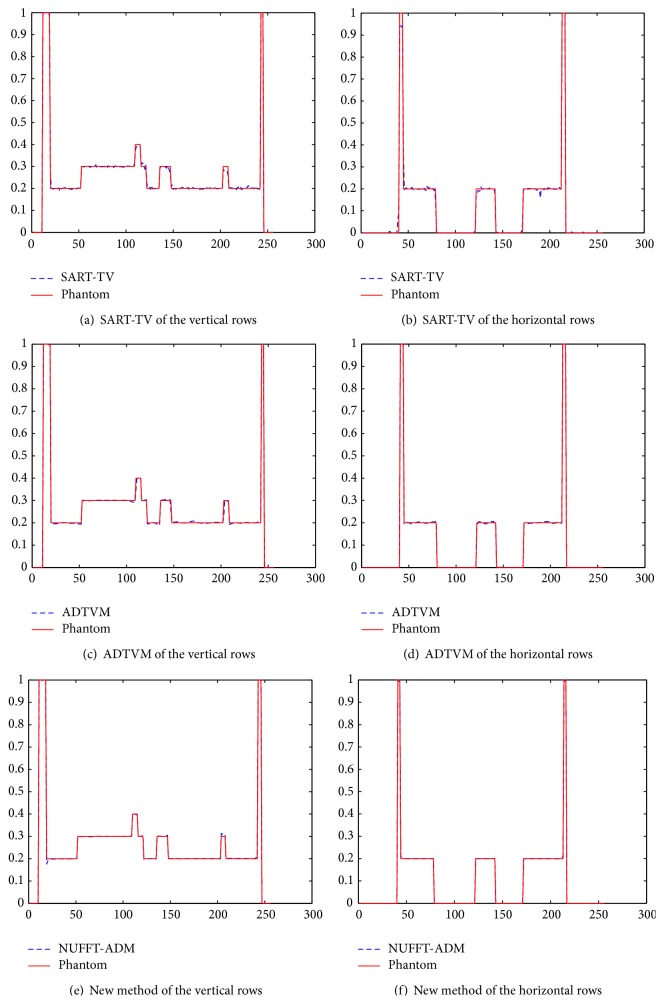
The image profile in Figure 1 shows (a) vertical profiles along the center of the SART-TV result, (b) horizontal profiles along the center of the SART-TV result, (c) vertical profiles along the center of the ADTVM result, and (d) horizontal profiles along the center of the ADTVM result. (e) Vertical profiles along the center of the NUFFT-ADM result and (f) horizontal profiles along the center of the NUFFT-ADM result.

To demonstrate the reconstruction accuracy quantitatively, the root-mean-squared error (RMSE) is used as a measurement of the reconstruction error. RMSE is defined as follows:

(19) $$\begin{array}{c} \text{RMSE}=\sqrt{{\frac{\sum_{i}\sum_{j}\left|f\left(i,j\right)-g\left(i,j\right)\right|}{N}}^{2}}, \end{array}$$

where *f* and *g* denote the ideal phantom and the reconstruction image, respectively; and *N* denotes the total number of pixels of the image. The image was reconstructed using SART-TV, ADTVM, the proposed method, respectively, and their results are presented in Figure 3. Two hundred iterations are performed for each algorithm. The profiles of these images along the central horizontal and vertical rows are shown in Figure 4 for the different methods.

**Figure 5:**
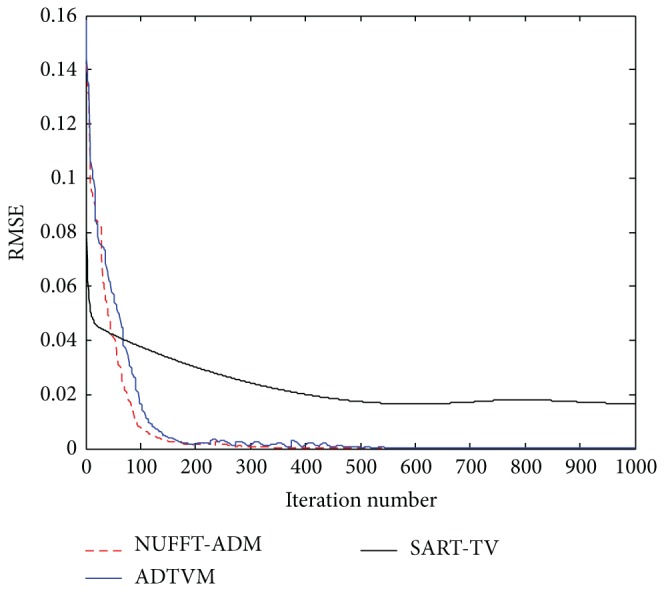
RMSEs as functions of iterations for three different algorithms.

RMSE is used as an evaluation criterion for different iteration times. The result is shown in Figure 5. The accuracy and running time of each reconstruction method at different iterations are presented in Table 2 for comparison.

The RMSEs, as well as the accuracy and running time of different methods, show that NUFFT-ADM significantly outperforms SART-TV and ADTVM. On one hand, the convergence of the new method is faster than that of SART-TV because of the use of the optimal solution with ADM. On the other hand, by taking advantage of the frequency NUFFT operator instead of the projection and back-projection in the spatial domain, which consumes the greatest amount of time among all components, NUFFT-ADM has a higher computation capability than the general algorithm in the spatial domain.

### 3.2. Reconstruction Using Real Data

To further verify the performance of the proposed algorithm, several experiments are performed to reconstruct a head model from real data using the new method. We compare the proposed algorithm with SART-TV and ADTVM.  Table 3 lists the scanning and reconstruction parameters. The detector elements are equidistantly spaced 0.635 mm from one another. The number of iterations for NUFFT-ADM, ADTVM, and SART-TV is 200.

**Figure 6:**
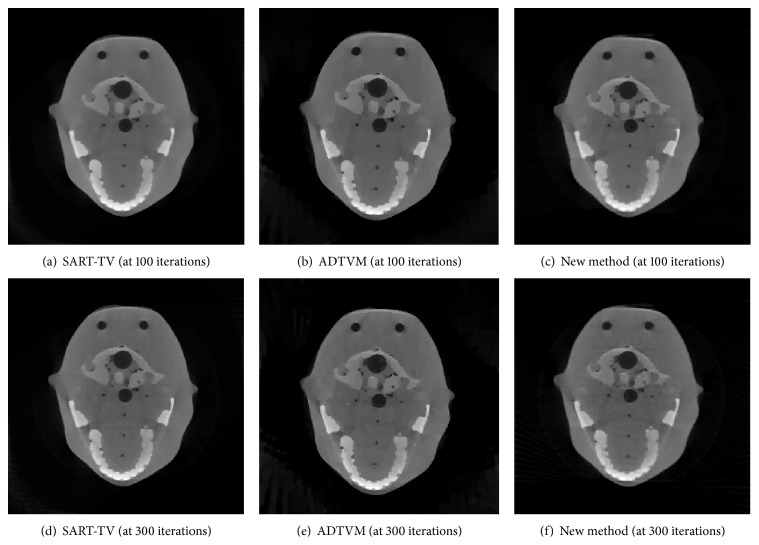
Reconstruction results of SART-TV, ADTVM, and NUFFT-ADM.

The reconstruction results are presented in Figure 6.

The reconstruction acquired results using real data clearly show that the quality of the reconstructed image is improved as the number of iterations is increased. Under the same number of iterations, the reconstruction results of NUFFT-ADM are superior to those of SART-TV, especially in terms of the high-frequency information that shows the image detail or volatile part. Compared to ADTVM, the detail of the image by the new method is nearly the same.

## 4. Conclusions

An optimal algorithm based on NUFFT for CT image reconstruction is presented in this work. The validity of the NUFFT-ADM algorithm is verified by conducting numerical simulations and real data experiments. The reconstruction results show that the proposed reconstruction algorithm improves reconstruction quality, accelerates convergence speed, and demonstrates lower computation complexity than other iterative algorithms. That is, the NUFFT-ADM algorithm can practically deal with fast image reconstruction from sparse projection measurements to reduce the radiation dose in X-ray CT. In principle, the proposed method can be extended to fan-beam geometry via the rebinning method to broad its application.

## Figures and Tables

**Table 1:** Parameters in the simulation of a sparse-view scan.

Parameters	Configuration
Detection elements	512
Source to axis distance	300 mm
Source to detection distance	600 mm
Views of projection data	18
Projection data	512 × 18
Reconstruction size	256 × 256 pixels
Pixel size	0.127 mm × 0.127 mm

**Table 2:** Accuracy and the running time of different method.

Iteration number	SART-TV		ADTVM		NUFFT-ADM	
	RMSE	Running time	RMSE	Running time	RMSE	Running time
100	0.0377	177.786 s	0.0165	107.439 s	0.0079	8.986 s
200	0.0302	349.544 s	0.0015	199.133 s	0.0012	16.752 s
500	0.0174	856.158 s	4.8927*e* − 4	508.376 s	1.6378*e* − 4	51.863 s

**Table 3:** Parameters in the simulation of a sparse-view scan.

Parameters	Configuration
Detection elements	640
Source to axis distance	678 mm
Source to detection distance	1610 mm
Views of projection data	60
Projection data	60 × 72
Reconstruction size	256 × 256 pixels
Pixel size	0.582 × 0.582 mm^2^
